# Access to Innovative Neurological Drugs in Europe: Alignment of Health Technology Assessments Among Three European Countries

**DOI:** 10.3389/fphar.2021.823199

**Published:** 2022-02-04

**Authors:** Lucia Gozzo, Giovanni Luca Romano, Serena Brancati, Marco Cicciù, Luca Fiorillo, Laura Longo, Daniela Cristina Vitale, Filippo Drago

**Affiliations:** ^1^ Clinical Pharmacology Unit/Regional Pharmacovigilance Centre, University Hospital of Catania, Catania, Italy; ^2^ Department of Biomedical and Biotechnological Sciences, University of Catania, Catania, Italy; ^3^ Department of Biomedical and Dental Sciences Morphological and Functional Images, University of Messina, AOU “G. Martino”, Messina, Italy; ^4^ Centre for Research and Consultancy in HTA and Drug Regulatory Affairs (CERD), University of Catania, Catania, Italy

**Keywords:** orphan drugs, neurological diseases, access, added therapeutic benefit, drug value

## Abstract

Even for products centrally approved, each European country is responsible for national market access after European Medicines Agency (EMA) approval. This step can result in inequalities in terms of access, due to different opinions about the therapeutic value assessed by Health Technology Assessment (HTA) bodies. This study aims to provide a comparative analysis of HTA recommendations issued by EU countries (France, Germany, and Italy) for new neurological drugs following EMA approval. In the reference period, we identified 11 innovative medicines authorized in Europe for five neurological diseases (cerebral adrenoleukodystrophy, spinal muscular atrophy, metachromatic leukodystrophy, migraine, and polyneuropathy in patients with hereditary transthyretin amyloidosis), including eight drugs for genetic rare diseases. We found no agreement on the therapeutic value (in particular the “added value” compared to the standard of care) of the selected drugs. Despite the differences in terms of assessment, the access has been usually guaranteed even if with various types of limitations. The heterogeneity of the HTA assessment of clinical data among countries is probably related to the uncertainties about clinical value at the time of EMA approval and the lack of long-term data and of direct comparison with available alternatives. Given the importance of new medicines especially for rare diseases, it is crucial to understand and act on the causes of inconsistency among the HTA assessments, in order to ensure rapid and uniform access to innovation for patients who can benefit.

## Introduction

According to recent data, neurology represents one of the therapeutic areas with the greatest number of development projects, perhaps reflecting scientific advances in the understanding of the basis of these diseases useful for potential novel intervention (Pankevich et al., 2014). Neurological conditions historically have been among the most difficult for which to develop effective and safe new therapies, due to the complexity in physiopathology and clinical presentation, and curative treatments for important diseases, such as neurodegenerative diseases, are still lacking (EC, 2020).

Actually, this is one of the most challenging therapeutic field in terms of likelihood of drug approval, with the longest time for review and recommendation (Arneric et al., 2018; Gribkoff and Kaczmarek, 2017; National Academies of Sciences, Engineering, and Medicine, 2016; O'Donnell et al., 2019).

A new drug (and/or an old drug for new indications) requires the authorization from a regulatory authority to be marketed (van Nooten et al., 2012; Gozzo et al., 2020a; Drago et al., 2020; Gozzo et al., 2021a; Toro et al., 2021). Moreover, price and reimbursement procedures need to be performed by competent authorities to find an agreement between companies and payers for market access (Gozzo et al., 2021a).

Today, in accordance with regulation 726/2004, in order to be marketed in the EU, the great majority of new, innovative medicines pass through a centralized procedure, which is compulsory for human medicines containing a new active substance to treat a lot of diseases, including neurodegenerative and rare diseases, for advanced-therapy medicinal products (ATMPs), and medicines derived from biotechnology processes in general (COMMUNITIES TCOTE, 1993; Regulation EC, 2004).

According to this procedure, the company submits a single marketing authorization (MA) dossier to the European Medicines Agency (EMA), and a MA for all the European Economic Area will be granted if the drug’s benefit–risk profile is positive according to the quality non-clinical and clinical data on safety and efficacy submitted by the applicant.

The aim of the centralized procedure is to enable rapid, EU-wide authorization of medicinal products (EMA, 2020a; Gozzo et al., 2020b; Gozzo et al., 2020c).

Despite the successful unification of the European procedures for drug approval, each country is responsible for national market access and pricing and reimbursement agreements, in line with national health needs and resources. This can result in access inequalities among European countries, due to differences not only in terms of willingness to pay but also in the recognition of drug therapeutic value (Ciani and Jommi, 2014; Gozzo et al., 2016; Akehurst et al., 2017; Allen et al., 2017). Indeed, in recent years, MA requests are submitted at earlier stages of development, especially for high-unmet medical need and/or rare diseases, before conclusive data are available, thus potentially leading to reduced quality of evidence and to uncertainty in terms of therapeutic value (Akehurst et al., 2017; Richardson and Schlander, 2019; Jommi et al., 2020; Brancati et al., 2021a; Brancati et al., 2021b).

The big challenge for policy makers is ensuring equitable access to medicines, balancing a timely patient access with the health system sustainability, in the era of precision medicines and advanced high-cost therapies (Drummond et al., 2008). The selection of medicines to be reimbursed is usually made by national Health Technology Assessment (HTA) bodies, based on cost-effectiveness, added value, and therapeutic need in the context of local standard of care (van Nooten et al., 2012; Angelis et al., 2018; Gozzo et al., 2021b).

In 2020, the EMA issued 78 positive opinions for new active substances (NASs), including eight medicines recommended for approval in the therapeutic area of neurology (10%) (EMA, 2020a).

This study aims to provide a review of the current evidence about innovative drugs for neurological diseases approved by the EMA in recent years and to perform a comparative analysis of HTA recommendations issued by EU countries for national pricing and reimbursement decisions.

## Methods

The study included the following steps:

1) Identification of new therapies with neurological indications approved in Europe between January 2011 and July 2021 listed on the registry published on the official EMA website (EMA, 2021a); we selected medicines of interest based on the Anatomical Therapeutic Chemical Classification (ATC) code N (NERVOUS SYSTEM, excluding drugs with exclusive psychiatric indication—ICD-10-CM Codes F01-F99) and M09 (OTHER DRUGS FOR DISORDERS OF THE MUSCULO-SKELETAL SYSTEM, to include drugs for neuromuscular disorders); generics and biosimilars were excluded, as well as those not representing a potential disease-modifying therapy (e.g., me-too drugs, namely, drugs structurally related to a first-in-class compound, belonging to the same therapeutic class, and used for the same therapeutic purposes);
2) Identification of the HTA assessments of drugs currently approved in Europe by the EMA performed by EU national authorities (France, Germany, and Italy); selection of countries was based on the availability of assessments for public consultation and on the clear definition of therapeutic values through comparable rating scales;
3) Comparative analysis of national opinions; available HTA reports and official administrative act of the three EU countries have been analyzed to compare the assessments.

Medicines centrally approved by the EMA have been identified by consulting the agency’s official documents and classified by type (e.g., gene therapy, small molecule, and monoclonal antibody), according to the orphan drug designation, and by type of authorization issued by the EMA (full, conditional, and for exceptional circumstances).

For each medicine, pivotal clinical trials were reviewed, analyzing the study design, the number of patients enrolled, the primary and secondary outcomes, and the main study results.

The level of clinical benefit (Service Médical Rendu—SMR) and the added therapeutic value compared to the available therapeutic alternatives (Amélioration du Service Médical Rendu—ASMR) was extracted from the official HTA documentation resulting from the assessment of the Transparency Committee (TC) of the French National Authority (Haute Autorité de santé—HAS) (SantèH-HAd, 2013; SantèH-HAd, 2014).

As regards Germany, we consulted the reports of the competent national bodies (Federal Joint Committee or Gemeinsamer Bundesausschuss, G-BA, and Institute for Quality and Efficiency in Health Care, IQWIG) containing a complete HTA on the additional therapeutic benefit of the product compared to recognized standard therapies (BundesausschussG-BG, 2010).

Finally, we identified the therapeutic need, the added therapeutic value, and the quality of the evidence from the Innovation Assessment Reports published by the Italian Medicines Agency (AIFA) (AIFADETERMINA DELL’AGENZIA ITALIANA DEL FARMACO, 2017). A direct comparison among national opinions was possible in terms of “added therapeutic value,” a measure included in all the available assessments (Supplementary Figure S1).

## Results

In the reference period, we identified 11 innovative medicines authorized in Europe (three gene therapies, two small molecules, three monoclonal antibodies, two antisense oligonucleotides, and one small interfering ribonucleic acid) for five for neurological diseases (cerebral adrenoleukodystrophy, spinal muscular atrophy, metachromatic leukodystrophy, migraine, and polyneuropathy in patients with hereditary transthyretin amyloidosis; Supplementary Table S1 and Table 1). Eight out of 11 medicines received orphan designation, all for genetic rare diseases. Only ATMP Zolgensma® received a conditional approval, whereas Vindaqel® was the only one approved under exceptional circumstances (Table 1).

**TABLE 1:** Innovative drugs with neurological indication approved in Europe in the reference period (2011–2021) and approval details.

	Active substance	ATC code	Type	Therapeutic indication	Conditional approval	Exceptional circumstances	Accelerated assessment	Orphan medicine	Marketing authorisation date
Skysona®	Elivaldogene autotemcel	N07	Gene replacement therapy	Treatment of early cerebral adrenoleukodystrophy in patients less than 18 years of age, with an *ABCD1* genetic mutation, and for whom a HLA-matched sibling HSC donor is not available	No	No	No	Yes	16/07/21
Evrysdi®	Risdiplam	M09AX10	Small molecule	Treatment of 5q SMA in patients 2 months of age and older, with a clinical diagnosis of SMA type 1, type 2, or type 3 or with 1–4 SMN2 copies	No	No	Yes	Yes	26/03/21
Libmeldy®	Atidarsagene autotemcel	N07	Gene replacement therapy	Treatment of MLD characterized by biallelic mutations in the ARSA gene leading to a reduction of the ARSA enzymatic activity: in children with late infantile or early juvenile forms, without clinical manifestations of the disease, and in children with the early juvenile form and with early clinical manifestations of the disease, who still have the ability to walk independently and before the onset of cognitive decline	No	No	Yes	Yes	17/12/20
Zolgensma®	Onasemnogene abeparvovec	M09AX09	Gene replacement therapy	Treatment of patients with 5q SMA with a biallelic mutation in the SMN1 gene and a clinical diagnosis of SMA type 1 or with 5q SMA with a biallelic mutation in the *SMN1* gene and up to three copies of the *SMN2* gene	Yes	No	No	Yes	18/05/20
Ajovy®	Fremanezumab	N02	Monoclonal antibody	Prophylaxis of migraine in adults who have at least four migraine days per month	No	No	No	No	28/03/19
Emgality®	Galcanezumab	N02	Monoclonal antibody	Prophylaxis of migraine in adults who have at least four migraine days per month	No	No	No	No	14/11/18
Onpattro®	Patisiran	N07	siRNA	Treatment of hATTR amyloidosis in adult patients with stage 1 or stage 2 polyneuropathy	No	No	Yes	Yes	27/08/18
Aimovig®	Erenumab	N02CX07	Monoclonal antibody	Prophylaxis of migraine in adults who have at least four migraine days per month	No	No	No	No	26/07/18
Tegsedi®	Inotersen	N07	ASO	Treatment of stage 1 or stage 2 polyneuropathy in adult patients with hATTR amyloidosis	No	No	Yes	Yes	06/07/18
Spinraza®	Nusinersen	M09	ASO	Treatment of 5q SMA	No	No	Yes	Yes	30/05/17
Vyndaqel®	Tafamidis	N07XX08	Small molecule	Treatment of transthyretin amyloidosis in adult patients with stage 1 symptomatic polyneuropathy to delayed peripheral neurologic impairment	No	Yes	No	Yes	16/11/11

Antisense oligonucleotide = ASO; arylsulfatase A = ARSA; haematopoietic stem cell = HSC; hereditary transthyretin-mediated amyloidosis = hATTR amyloidosis; human leukocyte antigen = HLA; metachromatic leukodystrophy = MLD; small interfering ribonucleic acid = siRNA; spinal muscular atrophy = SMA. Accelerated assessment is granted by the European Medicines Agency (EMA) for product of major interest for public health and therapeutic innovation. This procedure allows to reduce the timeframe for review a marketing-authorisation application (from up to 210–150 days). A conditional marketing authorisation may be granted with less comprehensive clinical data than normally required for medicines that address unmet medical needs, where the benefit outweighs the risk inherent in the fact that additional data are still needed. For this procedure, marketing approval is granted provided that the sponsor will provide missing data within an agreed timeframe. EMA may also grant a marketing authorisation under exceptional circumstances when comprehensive data cannot be obtained even after authorization, because the condition is rare or collection of full data is not possible or unethical.

In general, for all drugs (excluding Evrysdi® and Libmeldy®), data from phase II/III trials are available, almost half randomized, double blind, placebo controlled (Table 2). The median number of patients enrolled in these studies was 118 (range 6–1,949), followed for a median of 14 months (range 0.8–96).

**TABLE 2:** Data from clinical trials for innovative drugs approved in Europe in the reference period.

	Clinical trial	Study design	No. of patients	Primary outcome	Follow-up	Results
Skysona® (EMA, 2021b)	ALD-102	Open-label, single-arm prospective phase 2/3 study	32	Month 24 MFD-free survival (major functional disabilities) = loss of communication, cortical blindness, tube feeding, total incontinence, wheelchair dependence, or complete loss of voluntary movement	24 months	Twenty-seven out of 30 patients (90%, 95% CI: 73.5, 97.9) achieved month 24 MFD-free survival. Most patients (26/27, 96.3%) remained alive and maintained their MFD-free status through their last follow-up on study, including 14 patients with five or more years of follow-up
	ALD-104 (ongoing)	Open-label, single-arm phase 3 study	19 (35 planned)	Month 24 MFD-free survival (major functional disabilities) = loss of communication, cortical blindness, tube feeding, total incontinence, wheelchair dependence, or complete loss of voluntary movement	24 months	No subjects have completed the month 24 visit
Evrysdi® (EMA, 2021c)	BP39056 (FIREFISH)	Open-label, two-part study (part 1 was the dose-finding part of the study; part 2 was the confirmatory study)	21 (part 1)	The proportion of patients with the ability to sit without support for at least 5 s (sitting without support is never achieved in untreated patients with type 1 SMA)	24 months	After 12 months of treatment with risdiplam, 29.3% of patients in part 2 were sitting without support. This proportion is significantly higher than the pre-defined performance criterion of 5% based on natural history data (*p* < 0.0001)
			41 (part 2)			
	BP39055 (SUNFISH)	Part 1 was the exploratory dose-finding portion and part 2 was the randomized, double-blind, placebo-controlled confirmatory portion	51 (part 1)	Change from baseline score at month 12 on the Motor Function Measure-32 (MFM32)	12 months	The primary analysis for SUNFISH Part 2 showed a clinically meaningful and statistically significant difference between patients treated with Evrysdi and placebo. Change from baseline in MFM32 total score showed an improvement in the risdiplam group [change from baseline, LS means: 1.36 (95% CI: 0.61–2.11)], compared to a worsening observed in the PBO group [−0.19 (95% CI: 1.22, 0.84)]
			180 (part 2)			
Libmeldy® (EMA, 2021d)	Study 201222	Open-label, non-randomized, single-arm, prospective, comparative (non-concurrent control), phase I/II study	20	Co-primary endpoints: •Gross Motor Function Measure (GMFM): an improvement of >10% of the total GMFM score in treated patients, when compared to the GMFM scores in the age-matched, untreated historical control, evaluated at year 2 after treatment •ARSA activity: a significant (≥2 SD) increase in residual ARSA activity as compared to pre-treatment values, measured in peripheral blood mononuclear cells (PBMC) at year 2 after treatment	4.0 years (range 0.6–7.5 years)	Early-onset MLD patients treated before the onset of overt symptoms showed normal motor development, stabilization, or delay in the rate of progression of motor dysfunction as measured by GMFM total score
						A statistically significant increase in ARSA activity in PBMCs was also observed at year 2 post-treatment compared to pre-treatment baseline in both pre-symptomatic patients (20.0-fold increase; *p* < 0.001) and early symptomatic patients (4.2-fold increase; *p* = 0.004)
	Study 205756	Open-label, single-arm study	6	• Gross Motor Function Measure (GMFM)	0.87 years (range: 0.0–1.47 years)	Preliminary data on GMFM total score showed that gross motor function for all four subjects was within the range of gross motor function observed in a healthy cohort of children of similar chronological age. ARSA activity levels were detectable and within the normal range at month 3 in all three subjects with available data
				• ARSA activity		
Zolgensma® (EMA, 2020b)	CL-303	Phase III, open-label, single arm	22	Event-free survival (event = death or permanent ventilation)	18 months	90.9% (95% CI: 79.7%, 100.0%) event-free survival at 14 months
	CL-101	Phase I, open-label, dose-escalation	15	1 Requirement of respiratory assistance per day continuously for ≥2 weeks in the absence of an acute reversible illness, or	24 months	All treated patients had statistically significant improved survival without permanent ventilation
				2 Death		
	CL-302 (ongoing)	Phase III, open-label, single-arm	33	Achievement of developmental milestone	18 months	The primary efficacy endpoint independent sitting for at least 10 s at any time up to 18 months of age was met by six of the 32 patients (18.8%)
	CL-304 (ongoing)	Phase III, open-label, single-arm	At least 44 (as of the December 31, 2019 data cut-off, 29 patients were enrolled)	Achievement of developmental milestone	As of the efficacy data cut-off date of December 31, 2019, patients in cohort 1 had been in the study for an average of 10.5 months (range: 5.1–18 months). Patients in cohort 2 had been in the study for an average of 8.74 months (range: 2–13.9 months)	All patients in the study were alive and free of permanent ventilation at the data cut-off
Ajovy® (EMA, 2021e)	Study 1 (TEV-48125- 30050)	Randomized, double-blind, placebo-controlled phase III studies	875	Mean change from baseline in the monthly average number of migraine days	12 weeks	Both monthly and quarterly dosing regimens of fremanezumab demonstrated statistically significant and clinically meaningful improvement from baseline compared to placebo
	Study 2 (TEV-48125- 30049)	Randomized, double-blind, placebo-controlled phase III studies	1,130	Mean change from baseline in the monthly average number of headache days of at least moderate severity	12 weeks	Both monthly and quarterly dosing regimens of fremanezumab demonstrated statistically significant and clinically meaningful improvement from baseline compared to placebo
	Study 30051	Long-term study	Patients who completed the pivotal efficacy studies + approximately 300	Safety	15 months	For all episodic and chronic migraine patients, efficacy was sustained for up to 12 additional months. No safety signal was observed during the 15-month combined treatment period
Emgality® (EMA, 2021f)	EVOLVE-1	Phase 3, randomized, placebo-controlled, double-blind studies	843	The overall mean change from baseline in the number of monthly migraine headache days (MHDs)	6 months	Both galcanezumab 120 and 240 mg treatment groups demonstrated statistically significant and clinically meaningful improvements from baseline compared to placebo on mean change in MHD
	EVOLVE-2	Phase 3, randomized, placebo-controlled, double-blind studies	896	The overall mean change from baseline in number of monthly migraine headache days (MHDs)	6 months	Both galcanezumab 120 and 240 mg treatment groups demonstrated statistically significant and clinically meaningful improvements from baseline compared to placebo on mean change in MHD
	REGAIN	Phase 3, randomized, placebo-controlled, double-blind studies	1,085	The overall mean change from baseline in number of monthly migraine headache days (MHDs)	12 months	Both galcanezumab 120 and 240 mg treatment groups demonstrated statistically significant and clinically meaningful improvements from baseline compared to placebo on mean change in MHD
	Study CGAJ	Phase 3, long-term, randomized study	270	The overall mean reduction from baseline in the number of monthly MHDs	12 months	The overall mean reduction from baseline in the number of monthly MHDs averaged over the treatment phase was 5.6 days for the 120-mg dose group and 6.5 days for the 240-mg dose group
Onpattro® (EMA, 2021g)	APOLLO (ALN-TTR02-004)	Phase 3, randomized, double-blind, placebo-controlled study	225	Change from baseline in modified Neuropathy Impairment Score + 7 (mNIS + 7)	18 months	A statistically significant benefit in mNIS + 7 with Onpattro relative to placebo was observed at 18 months. Benefits relative to placebo were also observed across all mNIS + 7 components
	Study 003	Multicenter, phase 2, open-label, extension study	27	Mean change from baseline in the mNIS + 7	Up to 2 years	The mean change from baseline in the mNIS + 7 at 24 months was −6.95 (2.03) points
	Study 006	Multicenter, multinational, open-label extension study	184	Week 52 mNIS + 7	52 weeks	Week 52 mNIS + 7 efficacy data were available for 64 patients
Aimovig® (EMA, 2021h)	Study 20120295	Phase 2 randomized, multicenter, placebo-controlled, double-blind study	667	Change in mean monthly migraine days (MMD)	12 weeks	Reduction in mean monthly migraine days from placebo was observed in a monthly analysis from month 1, and in a follow-up weekly analysis, an onset of erenumab effect was seen from the first week of administration. Efficacy was sustained for up to 1 year in the open-label extension
					52-week open-label extension	
	Study 20120296	Phase 3, randomized, multicenter, placebo-controlled, double-blind study	955	Change from baseline in mean monthly migraine days	24-weeks	Patients treated with erenumab had a clinically relevant and statistically significant reduction from baseline in the frequency of migraine days from months 4–6 compared to patients receiving placebo. Efficacy was sustained up to 1 year in the active re-randomization part
					52-week active re-randomization part	
	Long-term follow-up study	Open-label treatment phase	383	–	5 years	Of the 383 patients, 168 (43.9%) discontinued with the most common reasons being patient request (84 patients; 21.9%), adverse events (19 patients; 5.0%), lost to follow-up (14 patients; 3.7%), and lack of efficacy (12 patients; 3.1%). The results indicate that efficacy was sustained for up to 5 years in the open-label treatment phase of the study
Tegsedi® (EMA, 2021i)	Pivotal study: CS2 (ISIS 420915-CS2)	Phase 2/3 multicenter, double-blind, placebo-controlled trial	172	Change from baseline in the modified Neuropathy Impairment Score + 7 tests (mNIS + 7) composite score and in the Norfolk Quality of Life–Diabetic Neuropathy (QoL-DN) questionnaire total score	Week 66	The changes from baseline in both primary endpoints (mNIS + 7 and Norfolk QoL-DN) demonstrated statistically significant benefit in favor of inotersen treatment at week 66. The differences were large with −19.73 (95% CI: 26.43, −13.03; *p* = 0.00000004) for the mNIS + 7 score (maximum score 346) and −11.68 (95% CI: 18.29, −5.06; *p* < 0.0006) for the Norfolk QoL-DN (maximum score 156)
	CS3 (ISIS 420915-CS3)	Phase 3 open-label extension study	114	Safety	5 years	The results obtained with the open-label extension study corroborated the results obtained with the CS2 study, and efficacy was maintained throughout the whole duration of the study
Spinraza® (EMA, 2021j)	Study CS3B (ENDEAR)	Phase 3, randomized, double-blind, sham-procedure-controlled study	121	Proportion of motor milestone responders	14 months	There were 21 (41%) subjects in the nusinersen group with a motor mile response at their last possible visit (day 183, 302, or 394 depending on the date they were treated), compared to 0/27 patients on control. This was highly statistically significant (*p* < 0.0001). In the final analysis, this percentage improved; 51% of subjects in the nusinersen group achieved a response compared to 0% in the control group (*p* < 0.0001)
				Time to death or permanent ventilation (≥16 h ventilation/day continuously for >21 days in the absence of an acute reversible event or tracheostomy)		There were 27/80 (34%) patients who died or required permanent ventilation on nusinersen compared to 20/41 (49%) on control. There were 12/80 (15%) deaths on nusinersen, compared to 13 (32%) on control. Overall, there was a 47% reduction in the risk of death or permanent ventilation compared to control: the risk of death was 62.8% lower in nusinersen-treated subjects than in those who received the sham procedure; the risk of permanent ventilation was 34% lower in nusinersen-treated subjects
	Study CS11 (SHINE)	Phase 3, open-label extension study	89 + 125	Number of participants experiencing adverse events (AEs) and/or serious adverse events (SAEs)	8 years	–
	Study CS3A	Open-label phase 2 study	20	Proportion of patients who improved in one or more categories in motor milestones	2 years	Twelve out of 20 patients (60%) in the study met the primary endpoint with improvement in mean motor milestone achievement over time
	Study CS4 (CHERISH)	Phase 3, randomized, double-blind, sham-procedure-controlled study	126	Change from baseline in Hammersmith Functional Motor Scale–Expanded (HFMSE) score	15 months	Subjects treated intrathecally with nusinersen achieved sustained and clinically meaningful benefits compared with a control group of subjects who received a sham procedure. A statistically significant change from baseline in HFMSE score was observed in the nusinersen group [4.0 (95% CI: 2.9–5.1)] compared to the sham control group [−1.9 (95% CI: 3.8–0.0)] (*p* = 0.0000002)
	Study CS7 (EMBRACE)	Phase 2, randomized, double-blind, sham-procedure study followed by a long-term open-label extension phase (part 2)	21	Number of participants with adverse events (AEs) and serious adverse events (SAEs)	Day 422	EMBRACE was terminated early due to positive results from other nusinersen trials, and patients were moved into the extension phase of EMBRACE (ongoing). Due to early termination, only six patients (43%) in the nusinersen group completed part 1 (assessment visit day 422), while none of the control group reached the 422 assessment visit day
	Study CS5 (NURTURE)	Phase 2, open-label, multicenter, single-arm study	17	Time to death or respiratory intervention (defined as invasive or non-invasive ventilation for ≥6 h/day continuously for ≥7 consecutive days or tracheostomy)	Efficacy data were available for 13 subjects at day 64, 10 subjects at day 183, and 5 subjects at day 302	No subjects died or had respiratory intervention (defined as either invasive or non-invasive ventilation for ≥6 h/day continuously for ≥7 consecutive days or tracheostomy)
Vyndaqel® (EMA, 2021k)	Study Fx-005	Phase II/III, multicenter, randomized, double-blind, placebo-controlled study	128	Neuropathy Impairment Score of the Lower Limb (NIS-LL—a physician assessment of the neurologic exam of the lower limbs) and the Norfolk Quality of Life–Diabetic Neuropathy [Norfolk QOL-DN—a patient-reported outcome, total quality of life score (TQOL)]	18 months	More tafamidis meglumine-treated patients were NIS-LL responders (change of less than 2 points on NIS-LL). Outcomes for the pre-specified analyses: at the primary timepoint (month 18), 45.3% of patients in the tafamidis group had an increase in the NIS-LL of <2, compared to 29.5% patients in the placebo group, but the differences between groups were not statistically significant (*p* = 0.068)
	Fx-006	Open-label extension study	71	Long-term safety and tolerability	12 months	The rate of change in the NIS-LL was similar to that observed in those patients randomized and treated with tafamidis in the previous double-blind 18-month period. The placebo-treated patients in the ITT population had progressively worse TQOL scores than tafamidis-treated patients, but the differences between groups were not statistically significant (7.2 versus 2.0, *p*-value = 0.1)
	Fx1A-201	Open-label, multicenter, single-arm study	21	Transthyretin stabilization at steady state, as measured by a validated immunoturbidimetric assay, in patients with non-V30M TTR amyloidosis	12 months	Treatment with tafamidis over 12 months in a mixed genotype population of patients with ATTR-PN resulted in TTR stabilization in 95% of patients by week 6 and 100% of patients at months 6 and 12, supporting persistence of TTR stabilization with chronic dosing of tafamidis

Table 3 reports the reimbursement status of the selected drugs. Except for the latest approved by the EMA (Skysona® and Libmeldy®), all drugs are reimbursed in the three EU countries.

**TABLE 3:** Reimbursement status in France, Germany, and Italy of neurological drugs approved by the EMA (x = reimbursed; / = not reimbursed or final opinion not available).

	Italy	France	Germany
Skysona®	–	–	–
Evrysdi ®	Compassionate use program (AIFA, 2021b)	x^d^ (HAS, 2021a)	x (G-BA, 2021a)
Libmeldy®	–	Authorization of early access (HAS, 2021b)	–
Zolgensma®	x^a^ ^e^ (AIFA, 2021c)	x^f^ (HAS, 2021c)	x (G-BA, 2021b)
Ajovy ®	x^a^ ^c^ (AIFA, 2021d)	x^h^ (HAS, 2021d)	x (G-BA, 2021c)
		Recommended reimbursement rate: 30%	
Emgality ®	x^a^ ^c^ (AIFA, 2021e)	x^h^ (HAS, 2021e)	x (G-BA, 2021d)
		Recommended reimbursement rate: 30%	
Onpattro®	x^a^ (AIFA, 2021f)	x (HAS, 2021f)	x (G-BA, 2021e)
		Recommended reimbursement rate: 65%	
Aimovig®	x^a^ ^c^ (AIFA, 2021g)	x^h^ (HAS, 2021g)	x (G-BA, 2021f)
		Recommended reimbursement rate: 30%	
Tegsedi ®	x^a^ (AIFA, 2021h)	x (HAS, 2021h)	x (G-BA, 2021g)
		Recommended reimbursement rate: 65%	
Spinraza®	x^a^ (AIFA, 2021i)	X^g^ (HAS, 2021j)	x (G-BA, 2021h)
Vyndaqel ®	x^a^ (AIFA, 2021j)	x (HAS, 2021i)	x (G-BA, 2021i)
		Recommended reimbursement rate: 100%	

a Eligibility criteria defined in the AIFA Registry (Bucolo et al., 2015; Fisichella et al., 2016; Gozzo et al., 2017; Breccia et al., 2020; Olimpieri et al., 2020; Breccia et al., 2021; Gozzo et al., 2021c).

b Stage 1 polyneuropathy in hATTR.

c Adults with at least 8 days of disabling migraine per month in the last 3 months and with insufficient response after at least 6 weeks of treatment or being intolerant or having clear contraindications to at least three previous classes of prophylaxis migraine drugs.

d Favorable opinion for reimbursement in the treatment of spinal amyotrophy 5q in patients aged 2 months and older with a clinical diagnosis of SMA, types 1, 2, and 3.

^e^Patients weighing up to 13.5 kg with clinical diagnosis of SMA type 1 and onset of symptoms in the first 6 months of life or with genetic diagnosis of SMA type 1 (biallelic mutation in the SMN1 gene and up to two copies of the SMN2 gene).

f Patients with spinal amyotrophy 5q (biallelic mutation of the SMN1 gene), with a clinical diagnosis of SMA types 1 and 2 or pre-symptomatic, having up to three copies of the SMN2 gene.

g Insufficient clinical benefit to justify reimbursement for 5q spinal muscular atrophy type IV.

h Favorable opinion for reimbursement in patients with severe migraine who have at least eight migraine days per month, with previous failure to at least two prophylactic treatments and without cardiovascular disease [patients having had a myocardial infarction, unstable angina, coronary artery bypass graft (CABG), percutaneous coronary intervention (PCI), stroke, deep-vein thrombosis (DVT), or other serious cardiovascular risk].

Data analysis showed that for 10/11 medicines, at least one public HTA evaluation from at least one of the three selected countries is available, and for six of these products, HTA reports have been published by all the three countries (Table 4). At the time of the analysis, no opinion has been published for Skysona®, the last medicine approved by the EMA. The highest score (important/considerable or major/maximum added value) has been recognized only by Italy (3/11, 27%; Zolgensma®, Onpattro®, and Spinraza®) and Germany (5/11, 45%; antibody for migraine, Onpattro® and Spinraza®).

**TABLE 4:** Agreement among opinions about therapeutic added value issued by member states.

	Italy	France	Germany
Skysona®	–	–	–
Evrysdi®	–	III^a^ (HAS, 2021a)	Hint of a non-quantifiable added benefit^b^ (IV)/additional benefit not proven (V)^c^ (G-BA, 2021a)
Libmeldy®	–	III^d^ (HAS, 2021b)	–
Zolgensma®	Important (II) (AIFA, 2021k)	III^e^/V^f^ (HAS, 2021c)	Additional benefit not proven (V) (G-BA, 2021b)
Ajovy ®	Low (IV) (AIFA, 2021L)	V (HAS, 2021d)	Additional benefit not proven^g^ (V)/hint for a considerable additional benefit^h^ (II) (G-BA, 2021c)
Emgality ®	Low (IV) (AIFA, 2021m)	V (HAS, 2021e)	Additional benefit not proven^g^ (V)/hint for a considerable additional benefit^h^ (II) (G-BA, 2021d)
Onpattro®	Important (II) (AIFA, 2021n)	III (HAS, 2021f)	Considerable additional benefit (II) (G-BA, 2021e)
Aimovig®	Low (IV) (AIFA, 2021o)	V (HAS, 2021g)	Additional benefit not proven^g^ (V)/h int for a considerable additional benefit^h^ (II) (G-BA, 2021f)
Tegsedi ®	–	IV (HAS, 2021h)	Non-quantifiable (IV) (G-BA, 2021g)
Spinraza®	Important (II) (AIFA, 2021p)	III/V^k^ ^(72)^	Major additional benefit^i^ (I)/Hint for a considerable additional benefit^j^ ^l^ (II) (G-BA, 2021h)
Vyndaqel ®	–	IV (HAS, 2021i)	Additional benefit not proven^m^ (V) (G-BA, 2021i)

a SMA, 2, like nusinersen, and SMA, 3 patients, not moving.

b Infantile form (SMA, 1) versus nusinersen.

c SMA, 2 and 3, and pre-symptomatic.

d Asymptomatic children without clinical manifestation.

e SMA, 1, pre-symptomatic with a genetic diagnosis of SMA (biallelic mutation of the SMN1 gene) and one to two copies of the SMN2 gene.

f SMA, 2,pre-symptomatic patients with a genetic diagnosis of SMA (biallelic mutation of the SMN1 gene) and three copies of the SMN2 gene.

g Untreated adult patients and patients who have responded inadequately to at least one prophylactic medication or are unable to tolerate or are unsuitable for at least one prophylactic medication or patients who are not responsive to or are unsuitable to or do not tolerate the medicinal therapies/active ingredient classes metoprolol, propranolol, flunarizine, topiramate, and amitriptyline.

h Patients who are not responsive to or unsuitable for or do not tolerate the medicinal therapies/active ingredients metoprolol, propranolol, flunarizine, topiramate, amitriptyline, valproic acid, or *Clostridium botulinum* toxin type A.

i 5q-SMA, 1 versus BSC.

j Pre-symptomatic children 5q-SMA, versus BSC.

k III, for SMA, 1 and 2 and pre-symptomatic infants and children with 5q SMA, with two–three copies of the SMN2 gene; V for SMA, 3.

l assessment updated on May 2021.

m assessment updated on May 2021’ comparing tafamidis to patisiran.

No agreements among the three EU states’ assessments were identified. German assessment was in accordance with the Italian one for Onpattro® and Spinraza®, with the French one for Tegsedi®, and at least in part for Zolgesma® and the three monoclonal antibodies for migraine.

## Discussion

In this study, we selected medicines recently approved by the EMA, which represent potential innovative treatment for neurological diseases, including gene therapies for rare genetic unmet medical needs.

Advanced therapies may provide significant health benefits generally with a single administration, allowing to act on the primary cause of a disease with the possibility of complete recovery and improvement of patient outcome potentially over the long term (Gozzo et al., 2021a).

Our results showed a lack of agreement on the therapeutic value (in particular the “added value”) of drugs recently approved for neurological indications in Europe. Despite the differences in terms of assessment, the access has been guaranteed in the three countries even if with various type of limitations.

Overall, the assessments issued by the German authorities were particularly positive, since the added therapeutic value has been classified as “major” or “considerable” in five cases out of 11 (45%), corresponding to five over nine drugs for which the evaluation has been made public (55%). Similarly, the AIFA granted the therapeutic value “important” for three drugs (3/11, 27%; 3/6 drugs for which the assessment has been made public to date, 50%), in particular in the case of treatments for spinal muscular atrophy (SMA) (Zolgensma® and Spinraza®) and of one treatment for hereditary transthyretin-mediated amyloidosis (hATTR) (Onpattro®). The Italian and German assessments were in accordance only for Spinraza® and Onpattro®. No drugs were judged to have a “major” or “important” added value according to HAS.

The quality of evidence supporting drug approval is undoubtedly a key point of the HTA process. Even if almost half of the studies are well-designed randomized, double-blind, placebo-controlled trials, it is noteworthy that no direct comparisons among the selected drugs with the same indication are available. This is one of the major issues for the HTA process management, especially with medicines approved earlier and earlier, since the lack of clear and robust evidence determines uncertainty about their therapeutic value and place in therapy.

In general, a direct comparison among drugs has been considered necessary for an adequate assessment of the additional benefit in Germany. For example, the G-BA considered the additional benefit of the ATMP Zolgensma® not proven, due to the lack of direct comparison with the available alternative nusinersen, and due to the limited clinical data available so far. Therefore, the German G-BA for the first time recommended to collect real-world evidence about Zolgensma® and Spinraza® through a registry study in order to close this evidence gap (Gozzo et al., 2021a; BundesausschussG-BG, 2021). Moreover, even for the third molecule approved for SMA, risdiplam, the G-BA concluded that no meaningful results are currently available, due to the lack of direct comparative data versus existing appropriate therapeutic alternatives, and recommended to collect data within the routine practice in order to improve the evidence for the benefit assessment.

Similarly, the French institutions considered that the lack of a direct comparison in clinical trials did not allow to clearly define the place in therapy of medicines for SMA (SantèH-HAd, 2020). However, in the absence of comparative data, in type 1 SMA and in pre-symptomatic patients with up to three copies of the *SMN2* gene, HAS considers Spinraza® and Zolgensma® as first-line treatments; Evrysdi® can be used as first line in symptomatic patients with type 1 SMA, but has no place in pre-symptomatic setting. The choice among these alternatives must be performed according to age, clinical status, comorbidities, different route of administration, and family choice. For example, the daily oral administration of risdiplam may be an attractive option compared to the other available modalities of administration but may not be suitable for the youngest children due to treatment compliance.

In type 2 SMA, Spinraza® and Evrysdi® are the treatment to be preferred, while in type 3, they represent the only therapeutic option.

On the contrary, the Italian agency explicitly accepted the possibility of having low-quality evidence in the case of rare and ultra-rare diseases (AIFADETERMINA DELL’AGENZIA ITALIANA DEL FARMACO, 2017), including the lack of a direct comparison with available alternatives. Indeed, in the case of Zolgensma®, the experts of the Italian Commission considered “important” the added value compared to the antisense oligonucleotide nusinersen (AIFA, 2021a), even with the limitations of the indirect comparison. Nevertheless, its use has been limited to a restricted population, specifically only in patients weighing up to 13.5 kg with clinical diagnosis of type 1 SMA and onset of symptoms during the first 6 months of life or with genetic diagnosis of SMA type 1 and up to two copies of the *SMN2* gene. Indeed, this subpopulation has been identified as the one with the greatest benefit and eligible to be reimbursed.

As regards to the monoclonal antibodies approved for migraine, the regulatory authorities of the three countries were in accordance with a low or no clinical added value in the management of the disease. In addition, the German institution delivered an opinion of “hint for a considerable additional benefit” of monoclonal antibodies compared to best supportive care (BSC), such as psychotherapy or relaxation techniques, only in adults who have at least four migraine days/month and for whom other substances used for prophylaxis (metoprolol, propranolol, flunarizine, topiramate, amitriptyline, valproic acid, or *Clostridium botulinum* toxin type A) have failed or have not been an option and BSC is the only treatment option.

Thus, limitations for the prescription of these drugs have been introduced, different among countries despite the overall agreement about the lack of added value.

A favorable opinion for reimbursement has been issued in France only in adults with severe migraine who have at least eight migraine days per month, after failure of at least two prophylactic treatments and without cardiovascular disease.

In Italy, the prescription can be performed according to the criteria of the AIFA Registry, in particular for adults with at least 8 days of disabling migraine per month in the last 3 months and with insufficient response after at least 6 weeks of treatment or being intolerant or having clear contraindications to at least three classes of prophylaxis migraine drugs.

According to the decision of the German GBA, a prescription is possible in patients with episodic migraine if *at least 5 substances* from the available pharmacological groups (beta-blockers, flunarizine, topiramate, valproic acid or amitriptyline) were not effective, not tolerated, or contraindicated (Hacke, 2020).

The variability in terms of regulatory decisions determining different patients access is probably related to the uncertainties about clinical value, the lack of long-term data and the demonstration of the superiority only versus placebo, as well as other non-clinical variables such as treatment cost.

In conclusion, the HTA process is a critical point for the assessment of drug value and patient access. Universally recognized clinical criteria for HTA recommendations include unmet medical needs, relative effectiveness, and safety of the new product compared to the available standard of care (van Nooten et al., 2012). The therapeutic added value versus available treatments should be one of the key determinants of patients access to innovative medicines. However, while relying on the evaluation of the same studies, a heterogeneity of the HTA assessment of clinical data has been observed among countries (Gozzo et al., 2021a). This heterogeneity, even beyond added value, does not necessarily translate into different reimbursement decisions, but often determines different eligibility criteria for patient treatment.

Given the importance of new medicines especially for rare and serious unmet needs, it is crucial to understand and act on the causes of inconsistency among the HTA assessments, in order to ensure rapid and uniform access to innovation for patients who can benefit.

In this context, the proposal for a regulation of the European Parliament and of the Council on health technology assessment amending the Directive 2011/24/EU drafted in 2018 and modified in 2021 aims to ensure a permanent cooperation on HTA at the EU level, sharing joint clinical assessments, joint scientific consultations, horizon scanning, and voluntary cooperation in non-clinical areas (European Commission and Directorate-General for Health and Food Safety, 2021). The adoption of this new regulation on HTA would be useful to harmonize HTA methodologies, hopefully leading to reduced disparities of medicine assessment among European countries.

## Data Availability

The original contributions presented in the study are included in the article/Supplementary Material, further inquiries can be directed to the corresponding author.
