# Supplementary material for: Access to Innovative Neurological Drugs in Europe: Alignment of Health Technology Assessments Among Three European Countries
Source: Front Pharmacol. 2022 Feb 4;12:823199. doi: 10.3389/fphar.2021.823199 (PMC8854989; doi:10.3389/fphar.2021.823199)

**Supplementary table 1.** drugs with neurological indication approved in Europe in the reference period (2011-2021)

| **N** | **Medicine name** | **Active substance** | **Therapeutic area** | **ATC code** | **Marketing authorisation date** |
| --- | --- | --- | --- | --- | --- |
| ***1*** | ***Skysona®*** | ***elivaldogene autotemcel*** | ***Adrenoleukodystrophy*** | ***N07*** | ***16/07/2021*** |
| 2 | Ontozry® | cenobamate | Epilepsy | N03AX | 26/03/2021 |
| 3 | Byfavo® | remimazolam | Conscious Sedation | N05CD | 26/03/2021 |
| ***4*** | ***Evrysdi®*** | ***Risdiplam*** | ***Muscular Atrophy, Spinal*** | ***M09AX10*** | ***26/03/2021*** |
| 5 | Fintepla® | Fenfluramine hydrochloride | Epilepsies, Myoclonic | N03 | 18/12/2020 |
| ***6*** | ***Libmeldy®*** | ***atidarsagene autotemcel*** | ***Leukodystrophy, Metachromatic*** | ***N07*** | ***17/12/2020*** |
| 7 | Exparel liposomal® | bupivacaine | Acute Pain | N01BB01 | 16/11/2020 |
| 8 | Zynrelef® | bupivacaine, meloxicam | Pain, Postoperative | N01B | 24/09/2020 |
| 9 | Gencebok® | Caffeine citrate | Apnea | N06BC01 | 19/08/2020 |
| ***10*** | ***Zolgensma®*** | ***onasemnogene abeparvovec*** | ***Muscular Atrophy, Spinal*** | ***M09AX09*** | ***18/05/2020*** |
| 11 | Sunosi® | solriamfetol hydrochloride | Narcolepsy; Sleep Apnea, Obstructive | N06BA14 | 16/01/2020 |
| 12 | Inbrija® | levodopa | Parkinson Disease | N04BA01 | 19/09/2019 |
| 13 | Epidyolex® | Cannabidiol | Lennox Gastaut Syndrome; Epilepsies, Myoclonic | N03AX | 19/09/2019 |
| 14 | Lacosamide UCB® | lacosamide | Epilepsies, Partial | N03AX18 | 26/08/2019 |
| 15 | Sixmo® | Buprenorphine hydrochloride | Opioid-Related Disorders | N07BC01 | 19/06/2019 |
| ***16*** | ***Ajovy®*** | ***fremanezumab*** | ***Migraine Disorders*** | ***N02*** | ***28/03/2019*** |
| 17 | Buvidal® | buprenorphine | Opioid-Related Disorders | N07BC01 | 20/11/2018 |
| ***18*** | ***Emgality®*** | ***Galcanezumab*** | ***Migraine Disorders*** | ***N02*** | ***14/11/2018*** |
| 19 | Kigabeq® | vigabatrin | Spasms, Infantile; Epilepsies, Partial | N03AG04 | 20/09/2018 |
| 20 | Slenyto® | melatonin | Sleep Initiation and Maintenance Disorders; Autistic Disorder | N05CH01 | 20/09/2018 |
| ***21*** | ***Onpattro®*** | ***patisiran sodium*** | ***Amyloidosis, Familial*** | ***N07*** | ***27/08/2018*** |
| ***22*** | ***Aimovig®*** | ***erenumab*** | ***Migraine Disorders*** | ***N02CX07*** | ***26/07/2018*** |
| ***23*** | ***Tegsedi®*** | ***inotersen sodium*** | ***Amyloidosis*** | ***N07*** | ***6/07/2018*** |
| 24 | Dzuveo® | sufentanil citrate | Pain | N01AH03 | 25/06/2018 |
| 25 | Zubsolv® | Buprenorphine hydrochloride, Naloxone hydrochloride dihydrate | Opioid-Related Disorders | N07BC51 | 10/11/2017 |
| ***26*** | ***Spinraza®*** | ***nusinersen sodium*** | ***Muscular Atrophy, Spinal*** | ***M09*** | ***30/05/2017*** |
| 27 | Ongentys® | opicapone | Parkinson Disease | N04 | 24/06/2016 |
| 28 | Wakix® | pitolisant | Narcolepsy | N07XX11 | 31/03/2016 |
| 29 | Briviact (in Italy: Nubriveo)® | Brivaracetam | Epilepsy | N03AX23 | 13/01/2016 |
| 30 | Zalviso® | sufentanil | Pain, Postoperative | N01AH03 | 18/09/2015 |
| 31 | Hetlioz® | tasimelteon | Sleep Disorders, Circadian Rhythm | N05CH | 3/07/2015 |
| 32 | Xadago® | safinamide methanesulfonate | Parkinson Disease | N04B | 23/02/2015 |
| 33 | Rasagiline ratiopharm® | rasagiline | Parkinson Disease | N04BD02 | 12/01/2015 |
| 34 | Duloxetine Lilly® | duloxetine | Neuralgia; Diabetic Neuropathies; Depressive Disorder, Major | N06AX21 | 8/12/2014 |
| 35 | Pregabalin Pfizer® | pregabalin | Anxiety Disorders; Epilepsy | N03AX16 | 10/04/2014 |
| 36 | Corbilta (previously Levodopa/Carbidopa/Entacapone Sandoz)® | levodopa, carbidopa, entacapone | Parkinson Disease | N04BA03 | 11/11/2013 |
| 37 | Selincro® | Nalmefene hydrochloride dihydrate | Alcohol-Related Disorders | N07BB05 | 24/02/2013 |
| 38 | Memantine Merz® | memantine hydrochloride | Alzheimer Disease | N06DX01 | 22/11/2012 |
| 39 | Fycompa® | perampanel | Epilepsies, Partial | N03AX22 | 23/07/2012 |
| **40** | ***Vyndaqel®*** | ***tafamidis*** | ***Amyloidosis*** | ***N07XX08*** | ***16/11/2011*** |
| 41 | Dexdor® | dexmedetomidine hydrochloride | Conscious Sedation | N05CM18 | 15/09/2011 |
| 42 | Buccolam® | midazolam | Epilepsy | N05CD08 | 4/09/2011 |
| 43 | Levodopa/Carbidopa/Entacapone Orion® | levodopa, carbidopa, entacapone | Parkinson Disease | N04BA03 | 23/08/2011 |
| 44 | Entacapone Orion® | entacapone | Parkinson Disease | N04BX02 | 18/08/2011 |
| 45 | Fampyra® | Fampridine | Multiple Sclerosis | N07XX07 | 20/07/2011 |
| 46 | Leganto® | rotigotine | Restless Legs Syndrome; Parkinson Disease | N04BC09 | 16/06/2011 |

**Supplementary table 2.** innovative drugs with neurological indication approved in Europe in the reference period (2011-2021); Antisense oligonucleotide = ASO; arylsulfatase A = ARSA; hereditary transthyretin-mediated amyloidosis = hATTR amyloidosis; human leukocyte antigen = HLA; metachromatic leukodystrophy = MLD; small interfering ribonucleic acid = siRNA; spinal muscular atrophy = SMA

| **N** | **Product** | **Type** | **Therapeutic indication** |
| --- | --- | --- | --- |
| ***1*** | ***Skysona®*** | Gene replacement therapy | Treatment of early cerebral adrenoleukodystrophy in patients less than 18 years of age, with an *ABCD1* genetic mutation, and for whom a HLA matched sibling haematopoietic stem cell donor is not available. |
| ***2*** | ***Evrysdi®*** | Small molecule | Treatment of 5q SMA in patients 2 months of age and older, with a clinical diagnosis of SMA Type 1, Type 2 or Type 3 or with one to four SMN2 copies |
| ***3*** | ***Libmeldy®*** | Gene replacement therapy | Treatment of MLD characterized by biallelic mutations in the ARSA gene leading to a reduction of the ARSA enzymatic activity:  - in children with late infantile or early juvenile forms, without clinical manifestations of the disease,  - in children with the early juvenile form, with early clinical manifestations of the disease, who still have the ability to walk independently and before the onset of cognitive decline |
| ***4*** | ***Zolgensma®*** | Gene replacement therapy | Treatment of patients with 5q SMA with a bi-allelic mutation in the SMN1 gene and a clinical diagnosis of SMA Type 1, or with 5q SMA with a bi-allelic mutation in the *SMN1* gene and up to 3 copies of the *SMN2* gene. |
| ***5*** | ***Ajovy®*** | Monoclonal antibody | Prophylaxis of migraine in adults who have at least 4 migraine days per month |
| ***6*** | ***Emgality®*** | Monoclonal antibody | Prophylaxis of migraine in adults who have at least 4 migraine days per month |
| ***7*** | ***Onpattro®*** | siRNA | Treatment of hATTR amyloidosis in adult patients with stage 1 or stage 2 polyneuropathy |
| ***8*** | ***Aimovig®*** | Monoclonal antibody | Prophylaxis of migraine in adults who have at least 4 migraine days per month |
| ***9*** | ***Tegsedi®*** | ASO | Treatment of stage 1 or stage 2 polyneuropathy in adult patients with hATTR amyloidosis |
| ***10*** | ***Spinraza®*** | ASO | Treatment of 5q SMA |
| ***11*** | ***Vyndaqel®*** | Small molecule | Treatment of transthyretin amyloidosis in adult patients with stage 1 symptomatic polyneuropathy to delay peripheral neurologic impairment |

**Supplementary figure 1.** *Grading of added therapeutic value according to national opinions*


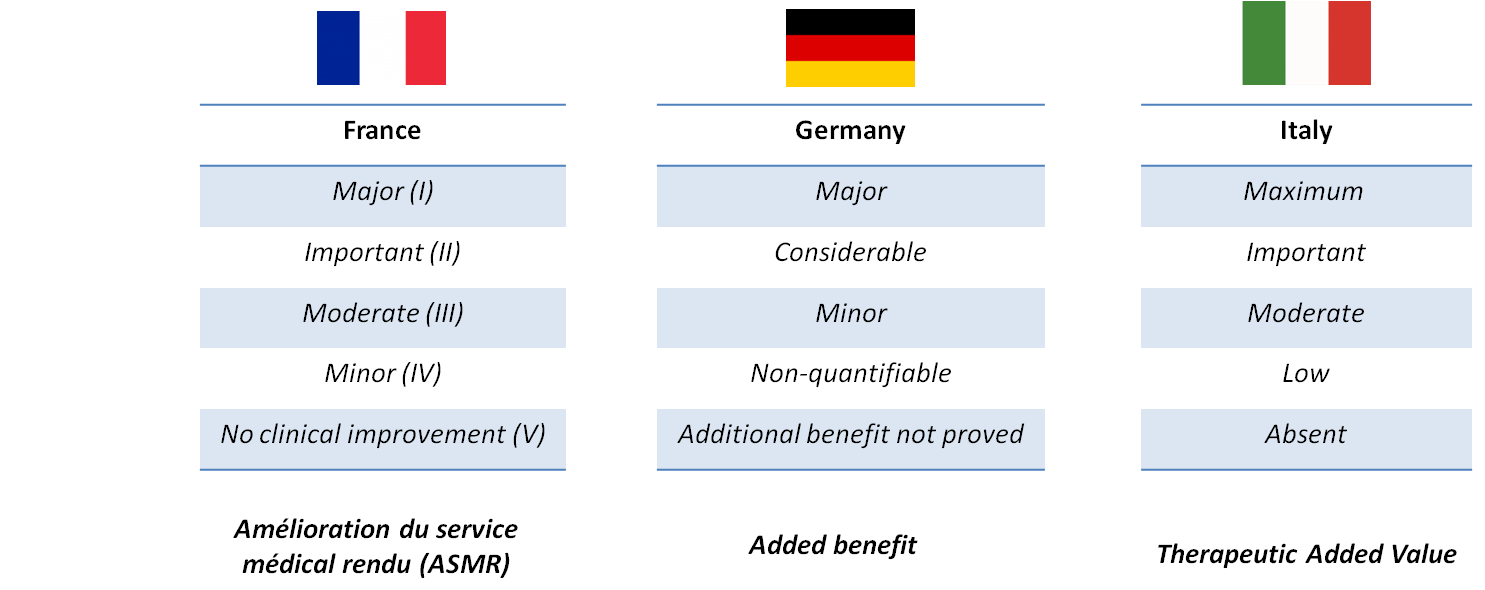

Supplement: Supplementary file 1 [file DataSheet1.docx]
